# Na^+^ and K^+^ transport and maturation stage ameloblast modulation

**DOI:** 10.3389/fphys.2023.1124444

**Published:** 2023-02-06

**Authors:** Jake Ngu, Antonius L. J. J. Bronckers, Kaitlin Katsura, Yan Zhang, Pamela K. Den Besten

**Affiliations:** ^1^ Department of Orofacial Sciences, University of California, San Francisco, San Francisco, CA, United States; ^2^ Department of Oral Cell Biology, Academic Centre for Dentistry Amsterdam (ACTA), MOVE Research Institute, University of Amsterdam and VU University, Amsterdam, Netherlands

**Keywords:** ameloblasts, KCNJ15, NCKX4, enamel, maturation, Kir4.2, Wdr72, fluorosis

## Abstract

**Introduction:** Enamel mineralization requires calcium transport into the extracellular matrix for the synthesis of hydroxyapatite (HA) crystals. Formation of HA releases protons into the matrix, which are then neutralized when ameloblasts modulate from cells with apical invaginations, the so-called ruffle-ended ameloblasts (RE), to smooth-ended ameloblasts (SE). Ameloblast modulation is associated with the translocation of the calcium exchanger Nckx4 to the apical border of RE, to remove Na^+^ from the enamel matrix in exchange for Ca^2+^ and K^+^. As enamel matures, Na^+^ and K^+^ in the matrix progressively decrease. However, the transporter to remove K^+^ from mineralizing enamel has not been identified.

**Methods:** Expression of K^+^ exchangers and channels in secretory and maturation stage of enamel organs were compared following an RNA-seq analysis. Kcnj15, which encodes the Kir4.2 inwardly rectifying K^+^ channel, was found to be the most upregulated internalizing K^+^ transporter in maturation stage of enamel organs. Kir4.2 was immunolocalized in wt, Nckx4^−/−^, Wdr72^−/−^, and fluorosed ameloblasts. Regulation of Wdr72 expression by pH was characterized *in vitro* and *in vivo*.

**Results:** Kir4.2 immunolocalized to the apical border of wild type (wt) mouse RE and cytosol of SE, a spatial distribution pattern shared by NCKX4. In Nckx4^−/−^ ameloblasts, Kir4.2 also localized to the apical surface of RE and cytosol of SE. However, in fluorosed and Wdr72^−/−^ ameloblasts, in which vesicle trafficking is disrupted, Kir4.2 remained in the cytosol. *In vitro*, Wdr72 was upregulated in LS8 cells cultured in medium with a pH 6.2, which is the pH of the enamel matrix underlying RE, as compared to pH 7.2 under SE.

**Conclusion:** Taken together these results suggest that Kir4.2 participates in K^+^ uptake by maturation ameloblasts, and that K^+^ and Na^+^ uptake by Kir4.2 and Nckx4, respectively, may be regulated by pH through WDR72-mediated endocytosis and membrane trafficking.

## Introduction

Enamel formation and mineralization is directed by epithelial derived ameloblasts and is unique among mineralizing tissues. Ameloblasts use ion-transporting transmembrane channels and transporters to deliver calcium and phosphate (Ca^2+^ and HPO_4_^2^) into the extracellular matrix to form hydroxyapatite (HA) crystals. These transporters include K^+^ -dependent Na^+^/Ca^2+^ Exchanger-4 (Nckx4) which co-transports Ca^2+^ and K^+^ into the enamel matrix in exchange for Na^+^ (Hu et al., 2012; Parry et al., 2013; Bronckers et al., 2017). Both Na^+^ and K^+^ pools in the enamel matrix are labile, and the decrease in both Na^+^ and K^+^ as enamel matures (Aoba and Moreno, 1987; Aoba et al., 1992; Lacruz, 2017), suggests that K^+^ transport into the matrix by Nckx4 is removed by an additional K^+^ transporter or channel.

Na^+^ and K^+^ in the maturing enamel of fluorotic mice (Lyaruu et al., 2014; Bronckers et al., 2015), is increased as compared to *wt* mice. Increased Na^+^ in fluorotic enamel is consistent with the finding that Nckx4 trafficking is disrupted in fluorosed ameloblasts (Bronckers et al., 2017). K^+^ accumulation in the enamel matrix of fluorosed mice suggests the possibility that intracellular transport of K^+^ is also affected by fluorosis.

Maturation-stage ameloblasts have been compared with parallel functions to the proximal renal tubule cells of the kidney (Lacruz et al., 2013), and K^+^ homeostasis is among the most important ions for maintaining cell function, particularly in regulating the electrochemical gradient across the plasma membrane (Palmer, 2015). To identify candidate K^+^ transporters and channels for the removal of extracellular K^+^ from the maturation enamel matrix, we compared transporters known to be important in kidney function, including Nkcc2, and Kcnj2 (ROMK), and genes coding for K^+^ channels in secretory as compared to maturation stage of enamel organs. We identified Kcnj15, encoding for Kir4.2 protein, as the most highly upregulated inwardly rectifying K^+^ channel. Kir4.2 (Kcnj15) immunolocalized to the apical border of wild type (*wt*) ruffle-ended ameloblasts (RE). In fluorosed and *Wdr72* ^*−/−*^ ameloblasts, where Nckx4 translocation to apical membrane is reduced (Wang et al., 2015; Bronckers et al., 2017), Kir4.2 remains primarily localized in the cytoplasm.

Expression of Wdr72*,* which is required for microtubule assembly and vesicle trafficking in maturation ameloblasts (Katsura et al., 2022), was upregulated in LS8 cells at acidic (pH 6.2), which is the pH of enamel matrix underlying RE. Together these results suggest that Kir4.2 (Kcnj15) removes K^+^ from the extracellular enamel matrix, associated with WDR72-mediated vesicle trafficking and extracellular matrix pH.

## Materials and methods

### Animals and tissue collection

C57BL/6 *wt*, *Nckx4* ^*−/−*^, *Wdr72* ^*−/−*^ mouse lines, and C57BL/6 female mice given 0 or 50 ppm fluoride drinking water for 5 weeks were housed and maintained in the UCSF animal care facility, which is an Associated for Assessment and Accreditation of Laboratory Animal Care (AAALAC) accredited barrier facility. All experimental procedures associated with these animal models were approved by the Institutional Animal Care and Use Committee (IACUC) under the protocol AN183449-03.

Postnatal 40-day (P40) mice were collected following standard IACUC protocols. Mice were anesthetized with 240 mg/kg tribromoethanol (Sigma-Aldrich), and following cervical dislocation hemimandibles were removed and fixed in 4% paraformaldehyde (PFA) for 24 h at 4°C. The hemimandibles were decalcified in 8% EDTA at 4°C for 3 weeks, with EDTA solution changes every other day, and then paraffin embedded and sectioned along their sagittal planes. Kidneys were collected from *wt* mice as a control tissue and either fixed for immunohistochemistry or frozen down for Western blot analysis.

### Enamel matrix element analysis

Hemimandibles from four 7-week old mice were dissected, embedded in epoxy, and polished in a cross orientation using a series of SiC papers and diamond polishing suspension starting from the incisor tip and stopping at the point of incisor eruption, near the plane of gingival sulcus. Six spots on the incisal enamel section at a plane perpendicular to incisal border of the lingual alveolar bone of each hemimandible were subjected to energy dispersive X-ray spectroscopy to collect elemental composition of K^+^ and Na^+^.

### RNA seq analyses

First molars from 20 pups at postnatal day 5 (P5, secretory stag) and 20 pups at day 12 (P12, maturation stage) were microdissected from mouse hemimandibles. Soft tissues were removed from the pulp chambers and enamel organs from five mice were pooled as a group, resulting in four groups for each time point. Total RNA was purified using Zymo Research RNA Miniprep Kit and then sent to Novogene Co for quality control assessment, cDNA library construction, labelling and sequencing. Differential expression analysis of two conditions was performed using the DESeq2 R package, and the resulting *p*-values were adjusted using the Benjamini and Hochberg’s approach for controlling the false discovery rate.

### Western blotting

First molars were microdissected from hemimandibles of eight P13 mice, and soft tissues were removed from pulp chambers. The molars were rinse with PBS, combined, and protein was extracted in Pierce™ RIPA buffer (Thermo Scientific, UC280926) with a proteinase inhibitor cocktail for mammalian cells (Millipore Sigma, P8340). These samples were frozen at −80°C for 30 min, and then centrifuged for 10 min at 4°C. The concentration of protein in supernatant was measured with Pierce™ BCA protein Assay kit (Thermo Scientific, 23225). Kidney tissues were similarly collected as a positive reference. Twenty micrograms of total proteins from each sample were mixed with equal amounts of 2X SDS reducing loading buffer. Samples were loaded onto 4%–20% Mini-PROTEAN TGX SDS-PAGE gels (Bio-Rad, 4561094) and were subjected to electrophoresis with Tris/glycine/SDS running buffer at 100 V for 90 min. Proteins on SDS-PAGE gels were transferred to nitrocellulose membranes using an Invitrogen iBlot™ Gel Transfer Device. Membranes were blocked with Intercept Protein Free Blocking Buffer (LI-COR, 927-90001) and then incubated overnight with rabbit anti-KCNJ15 antibody (Novus Biologicals, NBP1-83091) at 4°C. Following wash, blots were incubated with IRDye 680RD conjugated anti-rabbit IgG (LI-COR 926-68073) for 1 h at room temperature. The membranes were thoroughly washed with PBST and Kir4.2 protein with molecular mass of 42,577 Da was visualized by using an Odyssey XF Imaging System.

### Immunohistochemistry

Mouse hemimandible sagittal sections from *wt* mice were deparaffinized, rehydrated, and subjected to 10 mM sodium citrate buffer (pH 6.0) antigen retrieval for 3 h at 60°C. The sections were then treated with 1% acetic acid in PBS to inactivate endogenous activity and then blocked with GeneTex Universal Protein Blocking reagent for 1 h, followed by overnight incubation at 4°C with rabbit anti-SLC12A1 antibody (Sigma-Aldrich^®^ AV41388), rabbit anti-KCNJ1 antibody (Abcam ab224749), rabbit anti-KCNJ2 antibody (Abcam ab109750), or rabbit anti-KCNJ15 antibody (Novus Biologicals, NBP1-83091). After washing, sections were incubated with biotinylated swine anti-rabbit antibody (Dako E0431), followed by streptavidin, ALP (Vector^®^ SA-5100-1) for 1 h. Vector^®^ Red alkaline phosphatase substrate kit (Vector^®^ SK5100) used to visualize alkaline phosphatase labeled sections, followed by counterstaining with methyl green.

Mouse hemimandibles from *wt*, *Nckx4* ^*−/−*^, *Wdr72* ^*−/−*^ *,* and mice treated with 0 or 50 ppm fluoride for 5 weeks were similarly sectioned, treated with sodium citrate for antigen retrieval. Endogenous horseradish peroxidase activity was inactivated with 3% hydrogen peroxide in PBS, and then blocked with GeneTex Universal Protein Blocking reagent for 1 h, followed by overnight incubation at 4°C with either rabbit anti-KCNJ15 antibody (Novus Biologicals, NBP1-83091), or mouse anti-NCKX4 antibody (NeuroMab N414/25) (He et al., 2011). After washing, sections were incubated with anti-rabbit IgG HRP Conjugate (Promega W401). ImmPACT^®^ DAB Substrate Kit, Peroxidase (Vector^®^ SK-4105) was used to visualize HRP labeled sections and HRP, and stained sections were counterstained with hematoxylin, followed by dehydration and mounting. Slides were viewed under a Nikon Eclipse E800 microscope and images were taken with an Olympus DP74-CU camera.

### Cell culture and RT-qPCR

LS-8 cells were grown in 5% FBS/1% PS/DMEM to 80% confluence, washed with PBS one time, and then cultured overnight in MEM Eagle’s Media with 2% FBS/1% PS adjusted to pH 7.2 or pH 6.2. The cells were washed with PBS twice, then lysed with TRIzol™ Reagent (Ambion^®^, 15596018), and total RNA was purified using a Direct-zol™ RNA MiniPrep (Zymo Research, R2052) kit. The RNA concentration was quantified using a Thermo Scientific™ NanoDrop™ One Spectrophotometer, and then reverse transcribed into cDNA libraries using SuperScript™ III Reverse Transcriptase (Invitrogen™, 18080093) with 1500 ng of total RNA for each sample. qPCR was then performed using an Applied Biosystems 7500 Real-Time PCR System to quantitate the changes in the expression levels of *Wdr7*2 in cells grown at pH6.2 compared to that at pH 7.2 by delta delta Ct (ΔΔCt) method (Schmittgen et al., 2000; Bustin, 2002; Rao et al., 2013). The average Ct of *Wdr72* was obtained after normalizing to reference gene from a control experiment in which LS-8 cells were cultured in MEM Eagle’s Media with 2% FBS/1% PS at pH 7.2, was used as a baseline. Delta Ct of *Wdr72* expression levels of 5 separate experiments at pH 7.2 (ΔCt_pH7.2_) was obtained by normalizing the average Ct to this baseline. Then ΔΔCt was calculated by normalizing the average Ct of *Wdr72* expressed by cells grown at pH 6.2 in this same set of experiment (ΔCt_pH6.2_) to ΔCt_pH7.2_. The fold change was next calculated concerning 2^−ΔΔCt^. The significance was determined by unpaired two-tailed Student’s t-test. The fold changes from five experiments were graphed by GraphPad Prism Version 9.5.0.

Primer sequences were as follows:

*Gapdh* sense-5′-TGGCCTTCCGTGTTCCTAC-3′; anti-sense-5′-GAGTTGCTGTTG AAGTCGCA-3′

*Wdr72* sense-5′-TCTGGGGAAGAAAAGCTCCTC-3′; anti-sense-5′-CGAAGCCGAATGACCAAAAAG-3′

## Results

### Increased Na^+^ and decreased K^+^ are found in the enamel matrix of *Nckx4* ^*−/−*^ mice compared to *Nckx4* ^*+/+*^ mice

**FIGURE 1:**
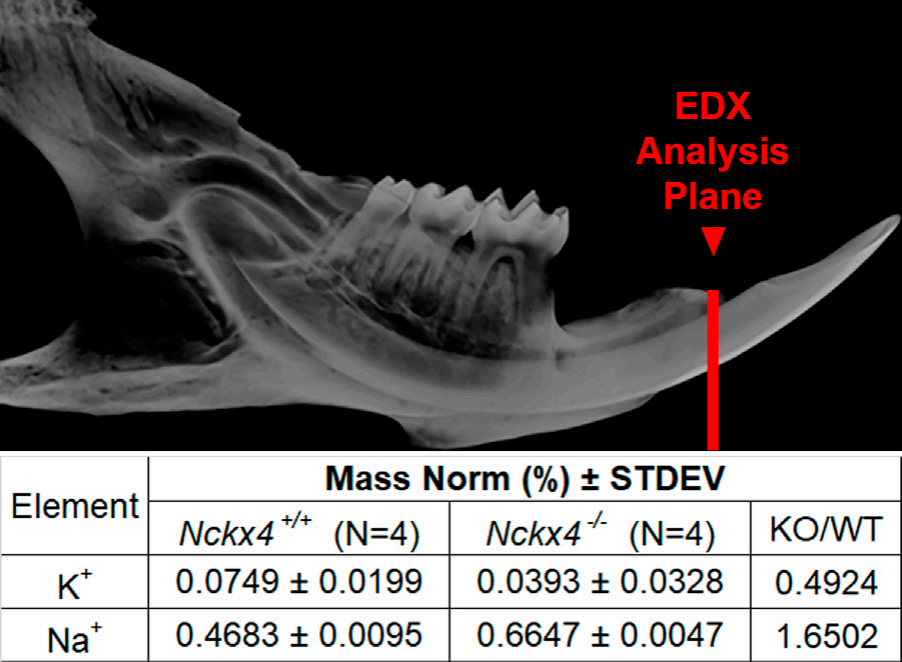
EDX analysis of *Nckx4* ^*+/+*^ and *Nckx4* ^*−/−*^ enamel. A red line drawn on a microCT image of a mouse mandibular hemimandible shows the plane of section analyzed by EDX. The mass norm % of K^+^ was decreased and Na^+^ increased in the *Nckx4* ^*−/−*^ enamel matrix relative to the *Nckx4* ^*+/+*^ matrix.

Nckx4 exchanges 1 K^+^ and 1 Ca^2+^ from the intracellular compartment, against 4 Na^+^ from the (extracellular) enamel fluid. As expected, energy dispersive X-ray (EDX) analysis showed reduced relative amounts of potassium by about 50%, while sodium increased about 60% in the *Nckx4* ^*−/−*^ enamel matrix as compared to that in the *Nckx4* ^*+/+*^ enamel (Figure 1).

### RNA-seq analysis identifies Kcnj15 as the most upregulated inwardly rectifying K^+^ transporter in the maturation stage of enamel organs

**FIGURE 2:**
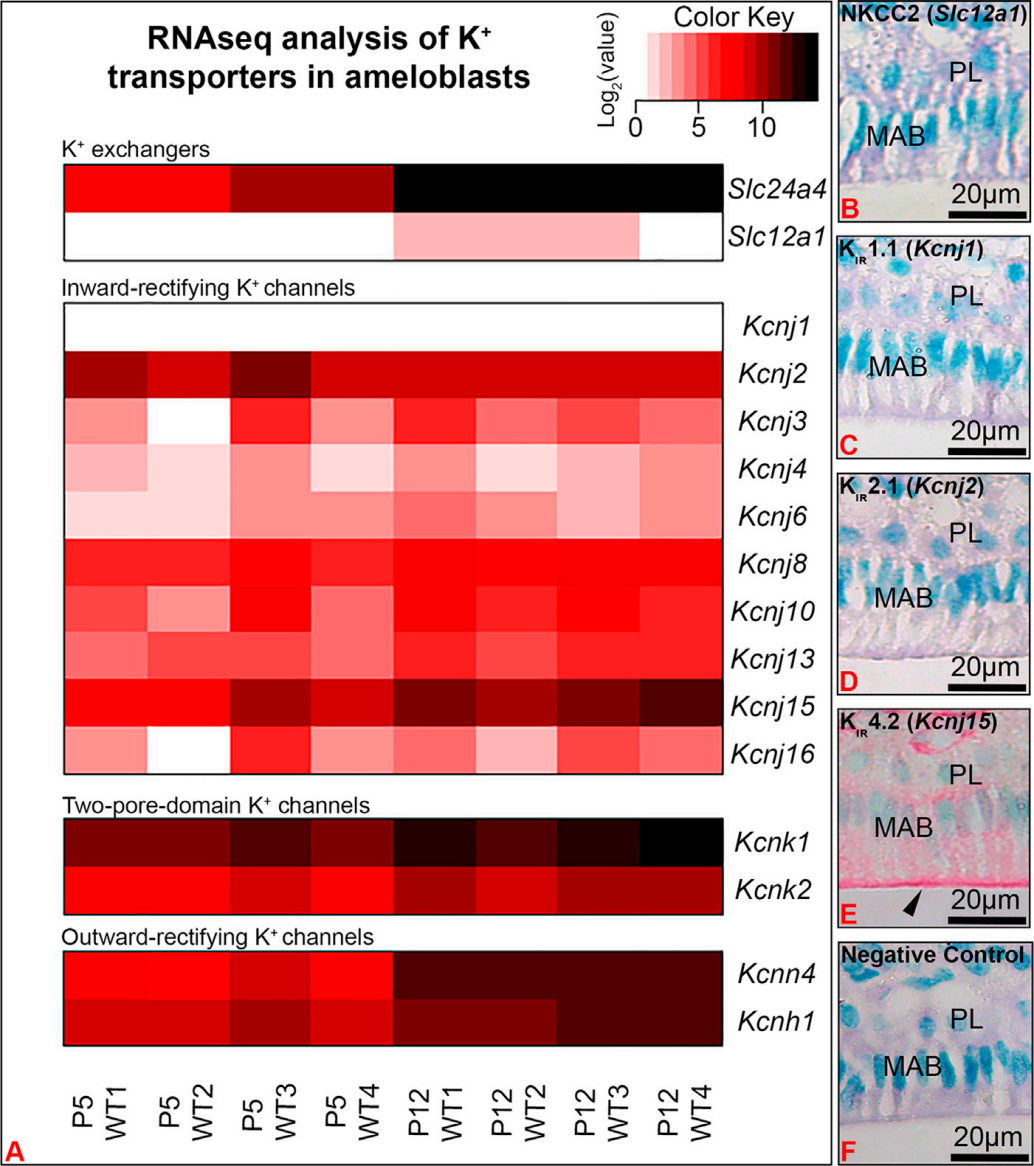
RNA-seq analysis showed Kcnj15 to be the most significantly upregulated inwardly rectifying K^+^ channel in maturation (P12) as compared to secretory (P5) enamel organs. **(A)** A heat map represents the expression levels of K^+^ transporters identified by RNA-seq in the P5 and P12 mouse first molar enamel organs. Immunohistochemical staining of NKCC2 (Slc12a1) **(B)**, Kir1.1 (Kcnj1) **(C)**, Kir 2.1(Kcnj2) **(D)** were immunonegative in maturation ameloblasts (MAB), while Kir4.2 (Kcnj15) was immunopositive, with increased staining at the ameloblast apical border. Negative control **(F)**. PL. papillary layer.

A heatmap of well-known major K^+^ transporters identified in our RNA-seq analyses confirmed that Slc24a4, the gene coding for Nckx4, was highly upregulated in maturation as compared to secretory enamel organs (Figure 2). Kcnj15, the gene encoding for the inwardly rectifying K^+^ channel Kir4.2 was the most highly upregulated inwardly rectifying K^+^ channel. Immunostaining showed Kir4.2 (Kcnj15), localized at the ameloblast apical border. K^+^ transporters found in the kidney, including Slc12a1, which encodes for Nkcc2, and Kcnj1, which encodes for Kir1.1 (ROMK1) (Palmer, 2015), were immunonegative in ameloblasts. Interestingly, the two-pore domain K^+^ channels Kcnk11 and Kcnk2, also called “leaky” and passive K^+^ channel, and the outwardly rectifying (transporting K^+^ from the intracellular to extracellular compartment) Kcnn4 and Kcnh1 were also upregulated in maturation as compared to secretory stage of enamel organs.

### Kir4.2 (Kcnj15) is synthesized by maturation stage enamel organ and spatially localizes at the ameloblast apical border

**FIGURE 3:**
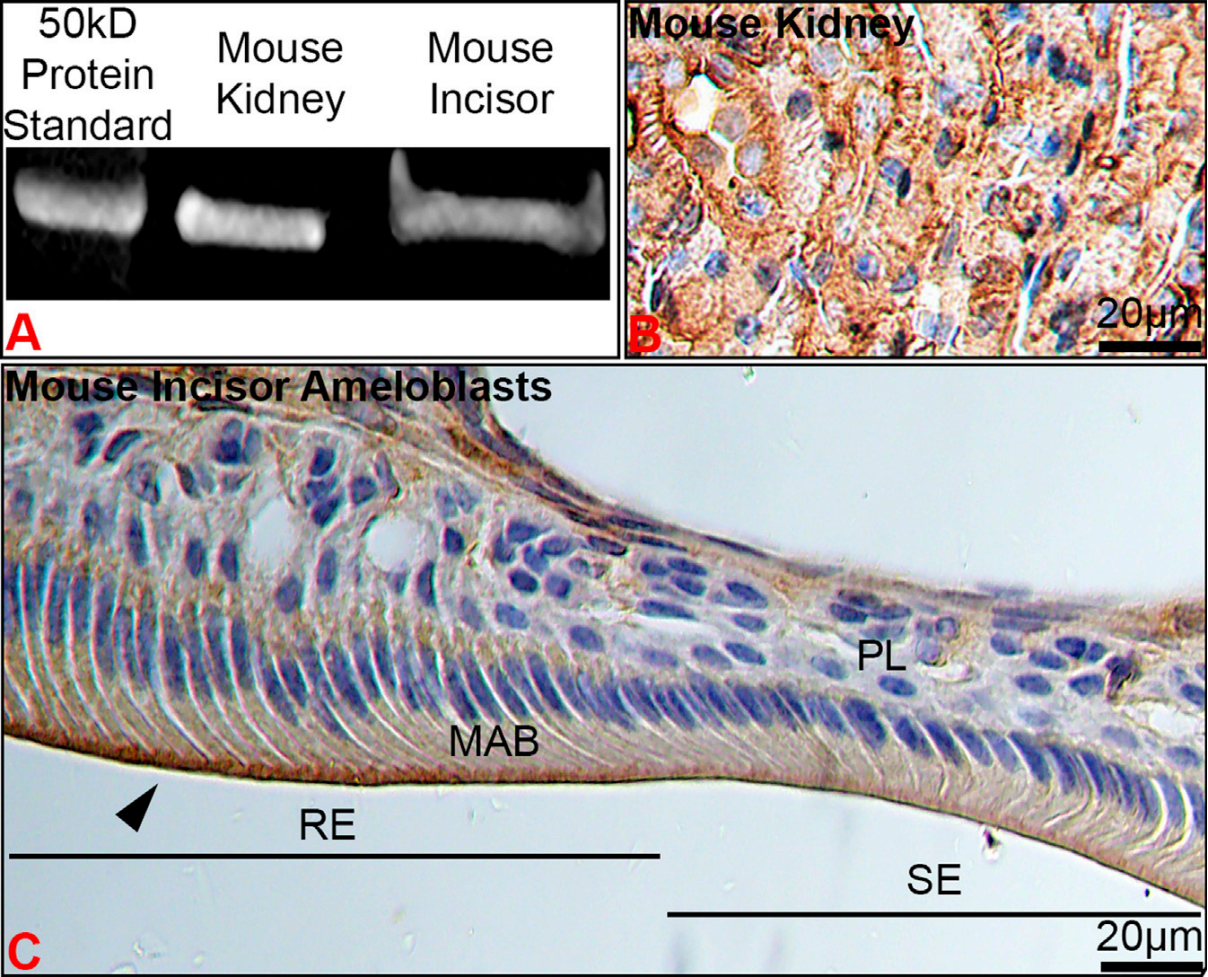
The stage- and spatial-specific presence of Kir4.2 (Kcnj15) in maturation ameloblasts. **(A)** Anti-Kir4.2 antibody identified an approximately 45 kDa protein in western blot analysis of mouse kidney and first molar enamel organs. **(B)** Kidney controls were immunopositive for Kir4.2. **(C)** Kir4.2 was immunopositive at the apical border of ruffle-ended (RE) maturation ameloblasts (MAB), and was reduced in smooth-ended (SE) ameloblasts.

To verify protein expression of Kir4.2, we performed Western blot and immunostaining of kidney and maturation stage ameloblasts on the continuously growing mouse incisor. Western blot showed Kir4.2 to be present in the proteins extracted from mouse first molar enamel organs, with a similar size to the Kir4.2 protein labeled in the kidney control (Figure 3A). Immunostaining of kidney validated the anti-Kir4.2 antibody (Figure 3B).

**FIGURE 4:**
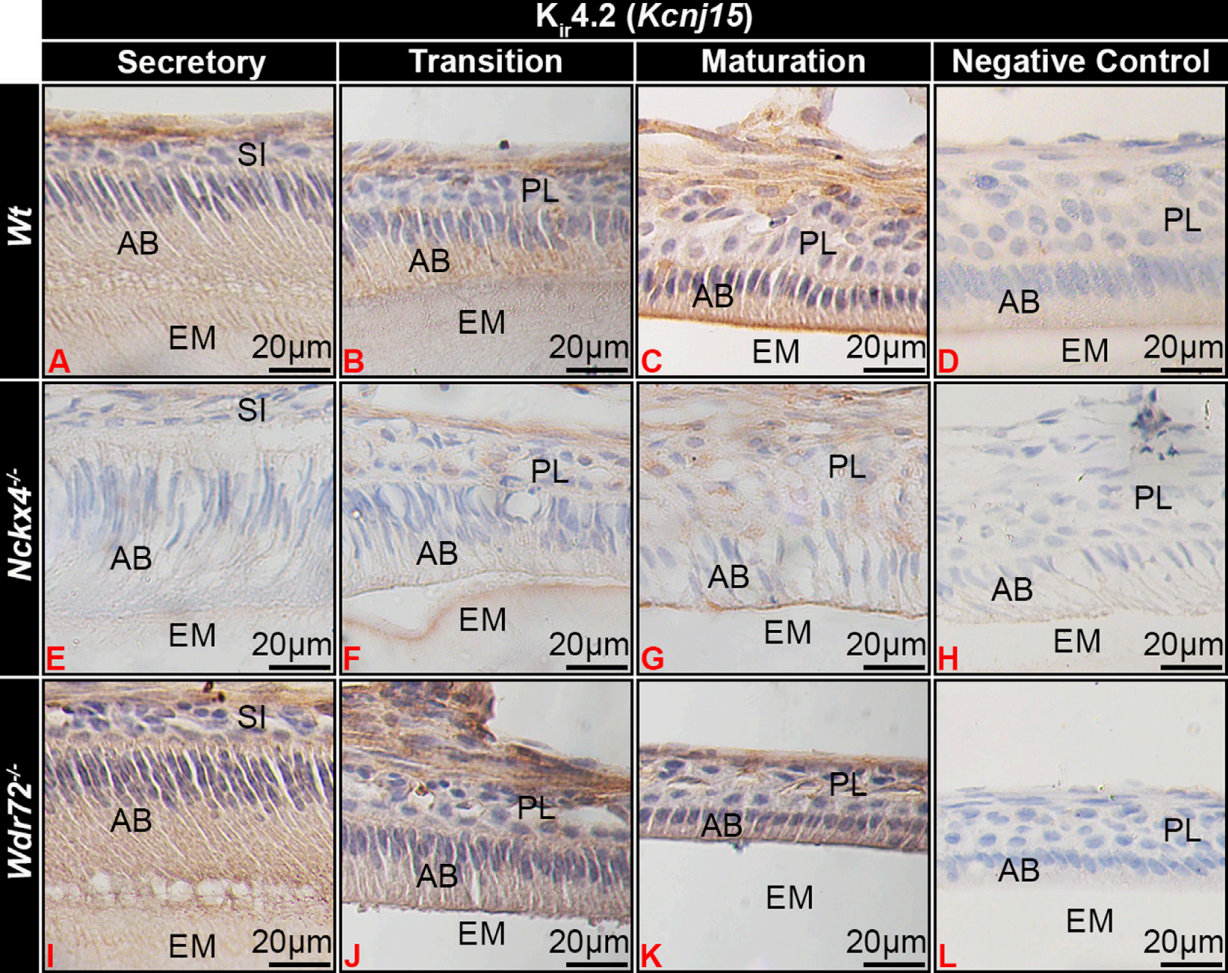
Kir4.2 immunostaining of ameloblasts. **(A)** *Wt* secretory ameloblasts (SAB) had minimal immunostaining for Kir4.2 (Kcnj15). **(B)** Transition stage ameloblasts (TAB) **(B)** had positive Kir4.2 cytoplasmic staining, which **(C)** localized to the apical border of ruffle-ended (RE) early-stage maturation ameloblasts (MAB) RE. In NCKX4 loss of function mice (*Nckx4* ^*−/−*^), Kir4.2 showed similar localization as compared to *wt* ameloblasts, with less intense immunostaining **(E–G)**. Intensity of Kir4.2 (Kcnj15) immunostaining in *Wdr72* ^*−/−*^ mice was similar to that of *wt* mice, but was not localized to the MAB apical border or *Wdr72* ^*−/−*^ mice **(I–K)**. Negative controls for MAB of each model *wt* **(D)**, *Nckx4* ^*−/−*^ **(H)**, and *Wdr72* ^*−/−*^ **(L)** were immunonegative. MAB, maturation ameloblast; EM, enamel matrix; PL, papillary layer.

In ameloblasts, Kir4.2 was enriched at the apical border of RE, whereas SE displayed a more diffuse pattern (Figures 3C, 4C). Positive immunostaining for Kir4.2 was also noted in the connective tissue cells around the dental epithelium and in cells that form walls of larger vessels near the epithelial papillary layer (Figures 2E, 3C).

### Kir4.2 is localized at the apical membrane of *Nckx4* ^*−/−*^ RA, but not in fluorosed or *Wdr72* ^*−/−*^ maturation ameloblasts

The phenotype of maturation stage ameloblasts of *Nckx4* ^*−/−*^ mice appeared to be similar to SE, but Kir4.2 also localized to the ameloblast apical border, though with apparent reduced staining intensity as compare to *wt* ameloblasts (Figures 4C, G). *Wdr72* ^*−/−*^ maturation ameloblasts showed similar intensity of Kir4.2 immunostaining as compared to wt ameloblasts, but remained in the cytoplasm of ameloblasts, without localizing to the apical plasma membrane (Figures 4I–K).

**FIGURE 5:**
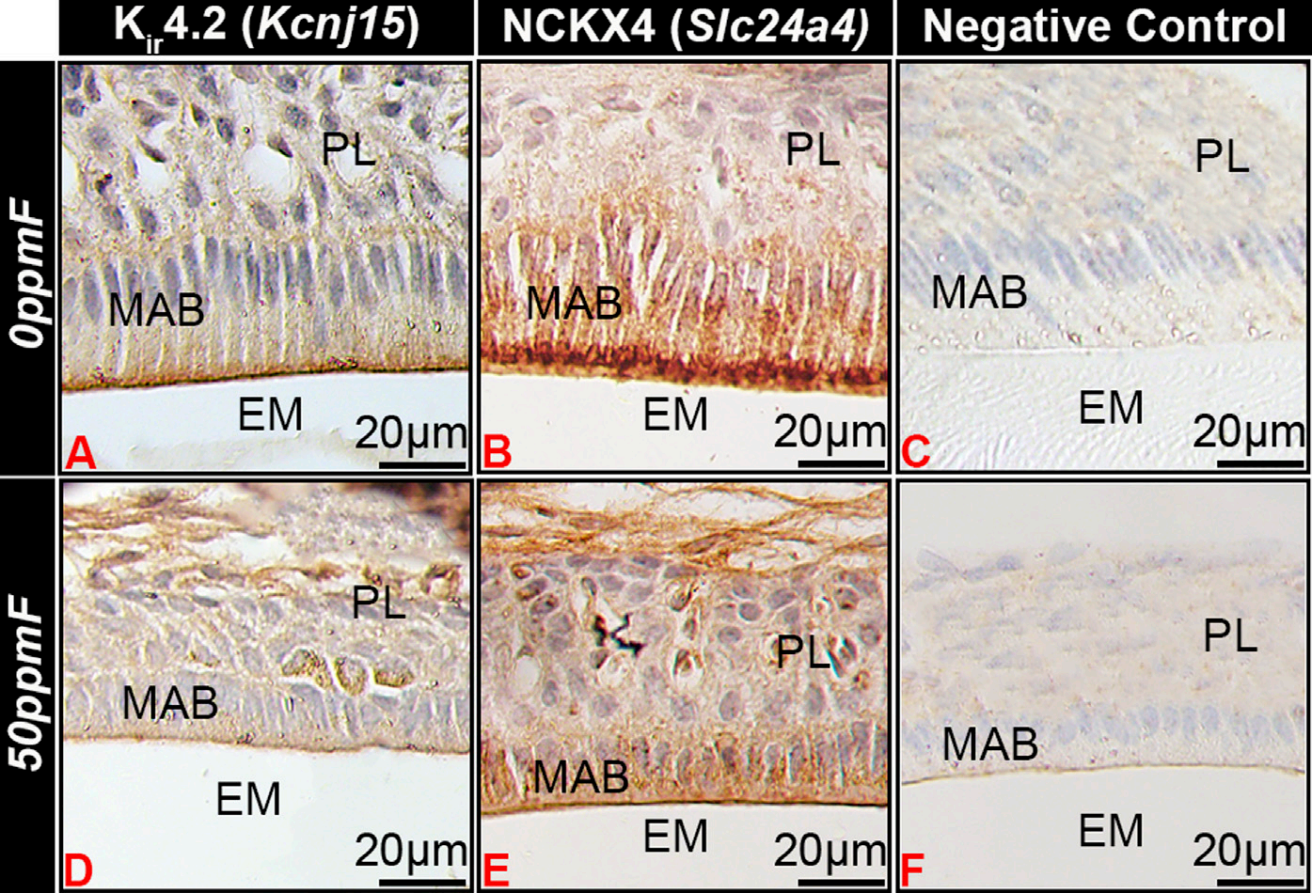
Fluorosis affects Kir4.2 (*Kcnj15*) localization in maturation ameloblasts. In mice given 0 ppm fluoride drinking water Kir4.2 (*Kcnj15* **(A)** and NCKX4 **(B)** localized to the apical border of ruffle-ended maturation stage ameloblasts. Fluorosed ameloblasts had reduced Kir4.2 (*Kcnj15*) **(D)** and NCKX4 **(E)** at the apical border as compared to 0 ppm fluoride controls. MAB, maturation ameloblast; EM, enamel matrix; PL, papillary layer.

In fluorosed ameloblasts, Kir4.2 immunostaining appeared reduced at the ameloblast apical border as compared to the 0 ppm fluoride control (Figures 5A, D) l, similar to similar NCKX4 (Figures 5B, E).

### The expression level of *Wdr72* is increased in LS8 cells grown at acidic pH (6.2) as compared to neutral pH (7.2)

**FIGURE 6:**
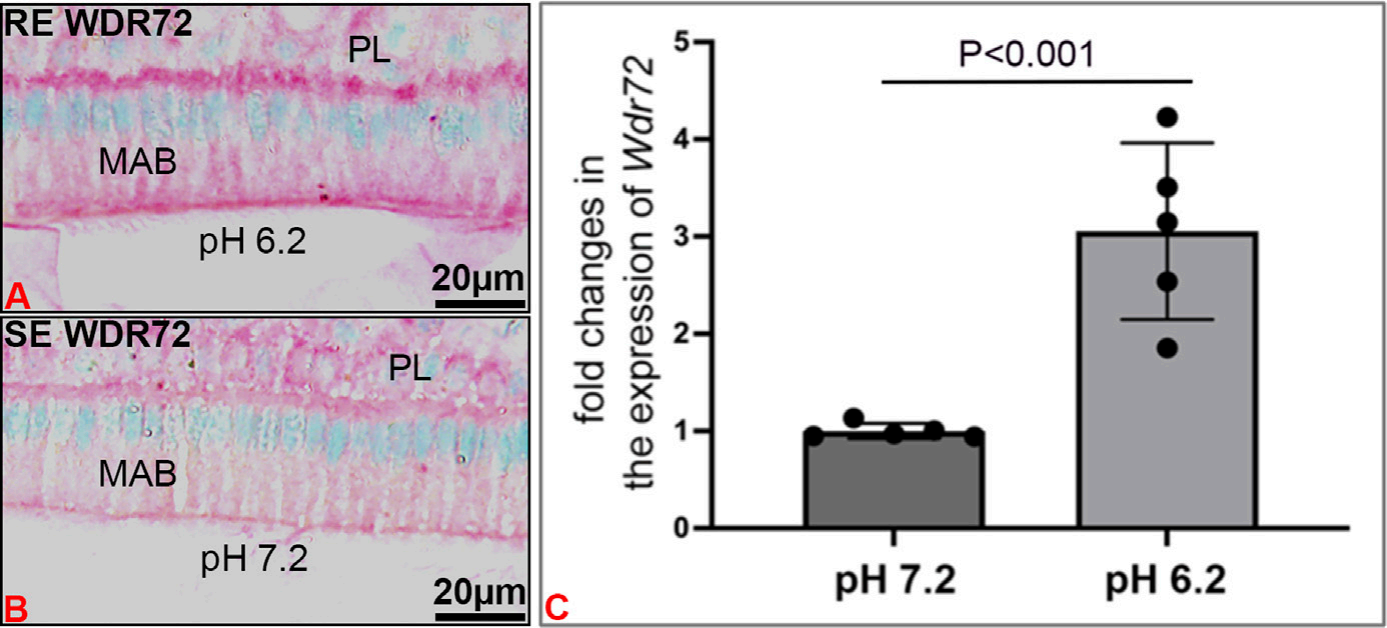
pH related effects on WDR72 expression. **(A)** Immunostaining of *wt* mouse incisor maturation stage of ameloblasts (MAB) showed increased staining of WDR72 at the basal and apical borders of ruffle-ended ameloblasts RE, associated with an acidic enamel matrix (pH 6.2), as compared to **(B)** Smooth-ended ameloblast SE which are associated with a more neutral matrix (pH 7.2) **(C)** *In vitro*, pH related changes in *Wdr72* mRNA showed increased expression at pH 6.2 as compared to pH 7.2 (unpaired Student’s t-test). *N* = 5; SDs of individual samples are indicated by the dark circles on the error bar.

*WDR72* immunostained at both the basal and apical borders, with more intense immunostaining at the apical border of RE, overlying enamel matrix with a pH of 6.2 (Figure 6A), as compared to SE which overlay an enamel matrix of pH 7.2 (Figure 6B). *In vitro*, relative expression of *Wdr72* in LS8 cells grown at pH 6.2 was significantly greater than in cells grown at pH 7.2 (Figure 6C).

## Discussion

As enamel matures, HA crystals expand in width, replacing the protein matrix. This process is driven by calcium transport to the extracellular matrix as ameloblasts modulate between cells with apical ruffled-ended borders (RE) and smooth-ended borders (SE) (Josephsen and Fejerskov, 1977). To support such large quantities of Ca^2+^ in a tightly regulated series of events, several ion exchangers that maintain Na^+^ and K^+^ homeostasis are key to functional enamel mineralization (Bronckers et al., 2015; Lacruz, 2017; Robertson et al., 2017). One such exchanger is NCKX4, which localizes to the apical border of RE and transports 1 K^+^ and 1 Ca^2+^ into the enamel matrix in exchange for 4 Na^+^. Consistent with this, we found that Na^+^ was relatively increased in *Nckx4* ^*−/−*^ as compared to *Nckx4* ^*+/+*^ maturation enamel, while K^+^ was relatively decreased in *Nckx4* ^*−/−*^ enamel.

**FIGURE 7:**
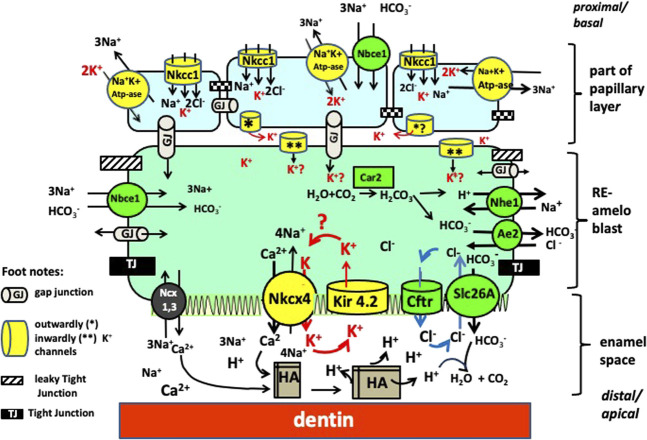
Working model for ion transport by RE ameloblasts. There are four clusters of transporters each involved in a specific function. By requiring the same ions to operate (Na^+^, K^+^ and Cl^−^) from the pool of ions these clusters are interdependent from each other. One cluster involves mineral transport to form crystals (NaPi2 and Nckx4 mediated transport of phosphate and calcium, respectively). A second cluster is for K^+^ transport (in yellow) to obtain electrochemical gradients to drive ion transport (Nckx4, Kir 4.2, Na^+^K^+^ ATpase, Nkcc1); Cluster 3 for pH and osmoregulation (in green, includes Cftr, Ae2, Nhe1 and members of the Slc26a family (SIc26a-a3-a4, a6, a7; Nhe1 and Car2). Cluster 4 for paracellular and intracellular transport tight junctions (black) and gap junctions (grey tubes).

Among the K^+^ transporter types expressed during enamel maturation, we found that Kcnj15, a gene encoding for the inwardly rectifying potassium channel, Kir4.2, to be relatively highly expressed in maturation stage ameloblasts. The Kir channels preferentially transport K^+^ intracellularly (Köhling and Wolfart, 2016), and inward rectification of K^+^ flux results from interactions between intracellular Mg^2+^ or polyamines, which physically block K^+^ outward flux by binding to residues localized in the transmembrane and cytoplasmic regions of the channels (Hibino et al., 2010). Other K^+^ channels, including the leaky two pore domain potassium channels, calcium-gated, and voltage-gated K^+^ channels, mainly conduct K^+^ outward. We therefore suggest that Kir4.2 (KCNJ15) at the apical surface, removes K+ from the labile pool of ions transported into the enamel matrix by NCKX4 (see Figure 7).

In *Wdr72* ^*−/−*^ and fluorosed ameloblasts, Kir4.2 remained localized in the cytoplasm. Kir proteins are transported by vesicle trafficking (Zangerl-Plessl et al., 2019), and altered vesicle trafficking, as evidenced by dislocation of Nckx4 in *Wdr72* ^*−/−*^ (Wang et al., 2015) and fluorosed (Bronckers et al., 2017) ameloblasts may have a role in translocation of Kir4.2 to the RE apical membrane. The lack and/or reduced translocation of Kir4.2 to the apical plasma membrane of fluorosed ameloblasts, may be why K+ is relatively increased in fluorosed enamel.

*Wdr72* modulates endocytosis and vesicle trafficking by affecting microtubule assembly (Katsura et al., 2022). Our finding that *Wdr72* expression was relatively increased at acidic pH, suggests that changes in enamel matrix pH could also affect vesicle trafficking by modulating WDR72 expression.

In fluorotic enamel there are fewer, but wider RE bands, suggesting a delayed transition from RE to SE (Smith et al., 1993). Fluorotic enamel contains less Cl^−^ (required for bicarbonate secretion into the enamel matrix) but more K^+^ (Lyaruu et al., 2014). Enamel of Cftr-null and Ae2-null, which have even less Cl^−^ and more K^+^ than fluorotic enamel, have no SE bands only RE bands(Bronckers et al., 2015). This suggests that local regulation of pH affects turnover of tight junctions.

Fluorotic enamel also has more Mg^2+^ than *wt* enamel, and furthermore, enamel of the more fluoride sensitive C57Bl mouse strain has more (approximately 2×) Mg^+^ in the extracellular enamel matrix as compared to the more fluoride resistant FVB mouse (Bronckers and Lyaruu, 2017). The role of Mg^2+^ in the regulation of K^+^ inward rectification by Kir channels may have a role in the sensitivity of ameloblasts to fluoride exposure. The mechanisms by which ameloblast control Mg transport are poorly understood, but likely to involve the cation channel TRPM7 (Nakano et al., 2016; Kádár et al., 2021).

**FIGURE 8:**
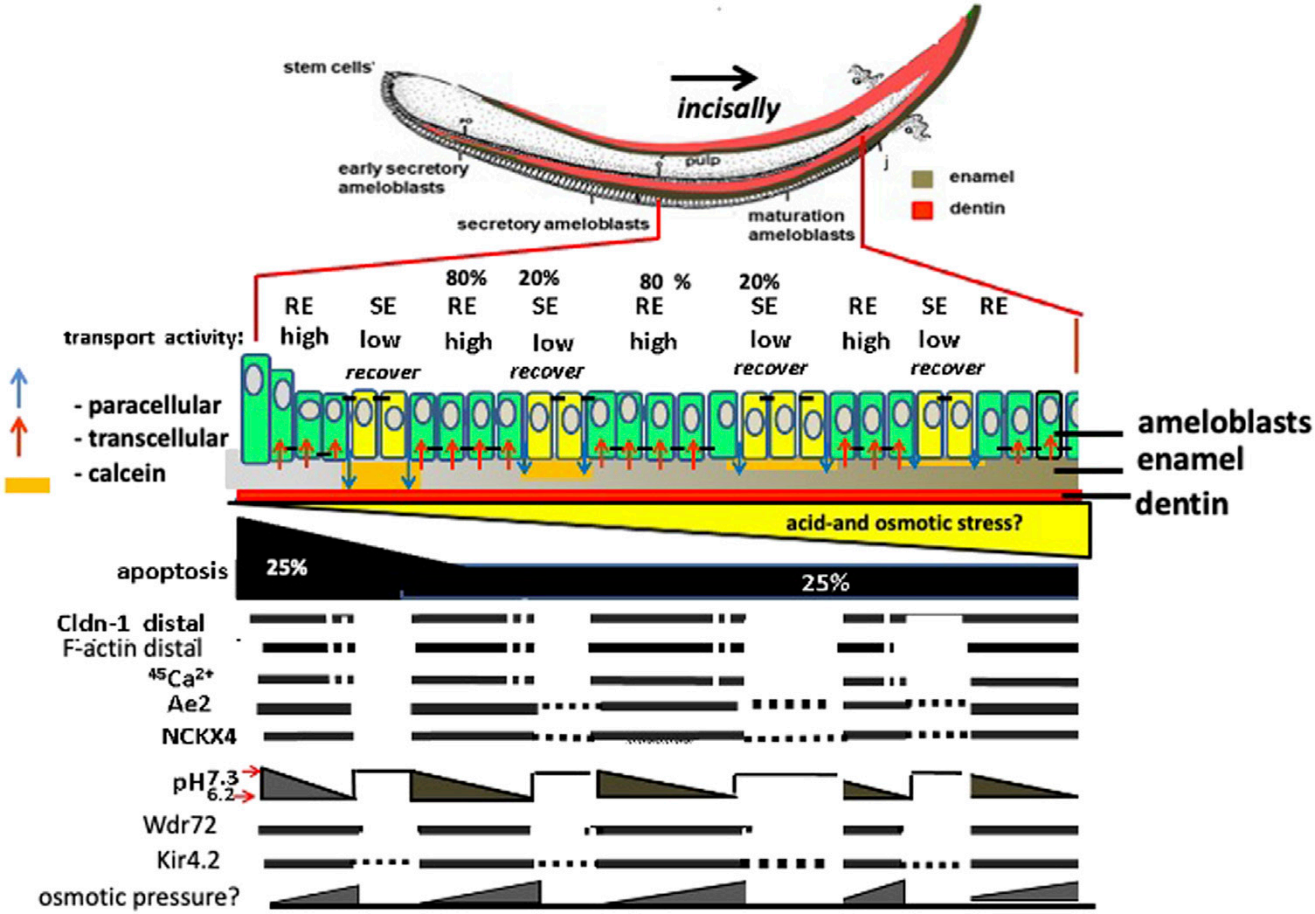
Amelogenesis in mouse incisor: major differences between expression/activity in RE compared to SE ameloblasts. Ameloblasts modulate corresponding to calcein staining of neutral pH enamel matrix orange color. Claudin 1 (Cldn1) and F-actin are increased in RE, associated TJs, and in RE, Nckx4 transports Ca^2+^ into the enamel matrix for hydroxyapatite (HA) formation. HA formation in enamel lowers the pH in enamel fluid. As matrix pH decreases, *Wdr72* increases, and NCKX4 and Kir4.2 translocate to the apical RE membrane. Paracellular transport of ions happens during transformation of RE into SE mode during which selective apical tight junctions disappear and basal tight junctions appear forming a new basal barrier. The transition of RE into SE and the flushing out of enamel fluid may result from hyperosmotic pressure of salt building up in the enamel space that causes leakage and eventually loss of the distal TJ by the inward flux of fluid.

Together these results support a role or Kir4.2 (Kcnj15) mediated inward K^+^ flux in maturation ameloblasts. Figure 8 illustrates the proteins and cellular changes associated with changes in RE and SE, and their relationship to TJ formation and ameloblast modulation. Future studies on the role of matrix pH, ion exchange, vesicle recycling, will further test this model and help to better understand the unique system by which maturation stage ameloblast direct enamel matrix mineralization.

## Data Availability

The data presented in the study are deposited in the GEO repository, accession number GSE222120.

## References

[B1] Aoba T. Moreno E. C. (1987). The enamel fluid in the early secretory stage of porcine amelogenesis: Chemical composition and saturation with respect to enamel mineral. Calcif. Tissue Int. 41, 86–94. 10.1007/BF02555250 3115550

[B2] Aoba T. Shimoda S. Moreno E. C. (1992). Labile or surface pools of magnesium, sodium, and potassium in developing porcine enamel mineral. J. Dent. Res. 71, 1826–1831. 10.1177/00220345920710111201 1401446

[B3] Bronckers A. L. Jalali R. Lytton J. (2017). Reduced protein expression of the Na+/Ca2++K+-Exchanger (SLC24A4) in apical plasma membranes of maturation ameloblasts of fluorotic mice. Calcif. Tissue Int. 100, 80–86. 10.1007/s00223-016-0197-4 27752731 PMC5215084

[B4] Bronckers A. L. Lyaruu D. Jalali R. Medina J. F. Zandieh-Doulabi B. Denbesten P. K. (2015). Ameloblast modulation and transport of Cl⁻, Na⁺, and K⁺ during amelogenesis. J. Dent. Res. 94, 1740–1747. 10.1177/0022034515606900 26403673 PMC4681480

[B5] Bronckers A. L. Lyaruu D. M. (2017). Magnesium, pH regulation and modulation by mouse ameloblasts exposed to fluoride. Bone 94, 56–64. 10.1016/j.bone.2016.10.014 27744011

[B6] Bustin S. A. (2002). Quantification of mRNA using real-time reverse transcription PCR (RT-PCR): Trends and problems. J. Mol. Endocrinol. 29, 23–39. 10.1677/jme.0.0290023 12200227

[B7] He W. Liu W. Chew C. S. Baker S. S. Baker R. D. Forte J. G. (2011). Acid secretion-associated translocation of KCNJ15 in gastric parietal cells. Am. J. Physiol. Gastrointest. Liver Physiol. 301, G591–G600. 10.1152/ajpgi.00460.2010 21719736 PMC3191558

[B8] Hibino H. Inanobe A. Furutani K. Murakami S. Findlay I. Kurachi Y. (2010). Inwardly rectifying potassium channels: Their structure, function, and physiological roles. Physiol. Rev. 90, 291–366. 10.1152/physrev.00021.2009 20086079

[B9] Hu P. Lacruz R. S. Smith C. E. Smith S. M. Kurtz I. Paine M. L. (2012). Expression of the sodium/calcium/potassium exchanger, NCKX4, in ameloblasts. Cells Organs 196, 501–509. 10.1159/000337493 PMC3535175 22677781

[B10] Jalali R. Lodder J. C. Zandieh-Doulabi B. Micha D. Melvin J. E. Catalan M. A. (2017). The role of Na:K:2Cl cotransporter 1 (NKCC1/slc12a2) in dental epithelium during enamel formation in mice. Front. Physiol. 8, 924. 10.3389/fphys.2017.00924 29209227 PMC5702478

[B11] Josephsen K. Fejerskov O. (1977). Ameloblast modulation in the maturation zone of the rat incisor enamel organ. A light and electron microscopic study. J. Anat. 124, 45–70. 914705 PMC1235513

[B12] Kádár K. Juhász V. Földes A. Racz R. Zhang Y. Löchli H. (2021). TRPM7-Mediated calcium transport in HAT-7 ameloblasts. Int. J. Mol. Sci. 22, 3992. 10.3390/ijms22083992 33924361 PMC8069123

[B13] Katsura K. Nakano Y. Zhang Y. Shemirani R. Li W. Den Besten P. (2022). WDR72 regulates vesicle trafficking in ameloblasts. Sci. Rep. 12, 2820. 10.1038/s41598-022-06751-1 35181734 PMC8857301

[B14] Köhling R. Wolfart J. (2016). Potassium channels in epilepsy. Cold Spring Harb. Perspect. Med. 6, a022871. 10.1101/cshperspect.a022871 27141079 PMC4852798

[B15] Lacruz R. S. (2017). Enamel: Molecular identity of its transepithelial ion transport system. Cell. Calcium 65, 1–7. 10.1016/j.ceca.2017.03.006 28389033 PMC5944837

[B16] Lacruz R. S. Smith C. E. Kurtz I. Hubbard M. J. Paine M. L. (2013). New paradigms on the transport functions of maturation-stage ameloblasts. J. Dent. Res. 92, 122–129. 10.1177/0022034512470954 23242231 PMC3545694

[B17] Lyaruu D. M. Medina J. F. Sarvide S. Bervoets T. J. Everts V. Denbesten P. (2014). Barrier formation: Potential molecular mechanism of enamel fluorosis. J. Dent. Res. 93, 96–102. 10.1177/0022034513510944 24170372 PMC3865793

[B18] Nakano Y. le M. H. Abduweli D. Ho S. P. Ryazanova L. V. Hu Z. (2016). A critical role of TRPM7 as an ion channel protein in mediating the mineralization of the craniofacial hard tissues. Front. Physiol. 7, 258. 10.3389/fphys.2016.00258 27458382 PMC4934143

[B19] Palmer B. F. (2015). Regulation of potassium homeostasis. Clin. J. Am. Soc. Nephrol. 10, 1050–1060. 10.2215/CJN.08580813 24721891 PMC4455213

[B20] Parry D. A. Poulter J. A. Logan C. V. Brookes S. J. Jafri H. Ferguson C. H. (2013). Identification of mutations in SLC24A4, encoding a potassium-dependent sodium/calcium exchanger, as a cause of amelogenesis imperfecta. Am. J. Hum. Genet. 92, 307–312. 10.1016/j.ajhg.2013.01.003 23375655 PMC3567274

[B21] Rao X. Huang X. Zhou Z. Lin X. (2013). An improvement of the 2ˆ(-delta delta CT) method for quantitative real-time polymerase chain reaction data analysis. Biostat. Bioinforma. Biomath. 3, 71–85. 25558171 PMC4280562

[B22] Robertson S. Y. T. Wen X. Yin K. Chen J. Smith C. E. Paine M. L. (2017). Multiple calcium export exchangers and pumps are a prominent feature of enamel organ cells. Front. Physiol. 8, 336. 10.3389/fphys.2017.00336 28588505 PMC5440769

[B23] Schmittgen T. D. Zakrajsek B. A. Mills A. G. Gorn V. Singer M. J. Reed M. W. (2000). Quantitative reverse transcription-polymerase chain reaction to study mRNA decay: Comparison of endpoint and real-time methods. Anal. Biochem. 285, 194–204. 10.1006/abio.2000.4753 11017702

[B24] Smith C. E. Nanci A. Denbesten P. K. (1993). Effects of chronic fluoride exposure on morphometric parameters defining the stages of amelogenesis and ameloblast modulation in rat incisors. Anat. Rec. 237, 243–258. 10.1002/ar.1092370212 8238976

[B25] Wang S. K. Hu Y. Yang J. Smith C. E. Nunez S. M. Richardson A. S. (2015). Critical roles for WDR72 in calcium transport and matrix protein removal during enamel maturation. Mol. Genet. Med. 3, 302–319. 10.1002/mgg3.143 PMC4521966 26247047

[B26] Zangerl-Plessl E. M. Qile M. Bloothooft M. Stary-Weinzinger A. Heyden M. A. G. (2019). Disease associated mutations in K(IR) proteins linked to aberrant inward rectifier channel trafficking. Biomolecules 9, 650. 10.3390/biom9110650 31731488 PMC6920955

